# Elution of Labile Fluorescent Dye from Nanoparticles during Biological Use

**DOI:** 10.1371/journal.pone.0025556

**Published:** 2011-10-06

**Authors:** Tiziana Tenuta, Marco P. Monopoli, JongAh Kim, Anna Salvati, Kenneth A. Dawson, Peter Sandin, Iseult Lynch

**Affiliations:** Centre for BioNano Interactions, School of Chemistry and Chemical Biology, University College Dublin, Belfield, Dublin, Ireland; The George Washington University, United States of America

## Abstract

Cells act as extremely efficient filters for elution of unbound fluorescent tags or impurities associated with nanoparticles, including those that cannot be removed by extensive cleaning. This has consequences for quantification of nanoparticle uptake and sub-cellular localization *in vitro* and *in vivo* as a result of the presence of significant amount of labile dye even following extensive cleaning by dialysis. Polyacrylamide gel electrophoresis (PAGE) can be used to monitor the elution of unbound fluorescent probes from nanoparticles, either commercially available or synthesized in-house, and to ensure their complete purification for biological studies, including cellular uptake and sub-cellular localisation. Very different fluorescence distribution within cells is observed after short dialysis times versus following extensive dialysis against a solvent in which the free dye is more soluble, due to the contribution from free dye. In the absence of an understanding of the presence of residual free dye in (most) labeled nanoparticle solutions, the total fluorescence intensity in cells following exposure to nanoparticle solutions could be mis-ascribed to the presence of nanoparticles through the cell, rather than correctly assigned to either a combination of free-dye and nanoparticle-bound dye, or even entirely to free dye depending on the exposure conditions (i.e. aggregation of the particles etc). Where all of the dye is nanoparticle-bound, the particles are highly localized in sub-cellular organelles, likely lysosomes, whereas in a system containing significant amounts of free dye, the fluorescence is distributed through the cell due to the free diffusion of the molecule dye across all cellular barriers and into the cytoplasm.

## Introduction

Understanding the interactions between nanoparticles and living systems is vital to realisation of the enormous potential impact of nanotechnology on health, medicine and the environment. Thus, we need to understand how nanoparticles enter organisms, tissues, and cells; where they go when they get there, and the consequences of them being there (the biological impacts). In order to make the connection between nanoparticle uptake, sub-cellular localisation and biological impacts it is necessary to be able to visualise the uptake of the nanoparticles by the living systems, and this typically requires labelling of some description in order to utilise the advanced biological techniques that have emerged in recent years, such as confocal fluorescence microscopy and live cell imaging.

The present work shows that the challenges inherent in the preparation of labelled nanoparticles for uptake and other biological studies are significantly greater than previously understood, as issues such as label brightness and elution of the label in physiological milieu can complicate the interpretation of nanoparticle uptake and sub-cellular distribution results. This has recently been shown to apply to many of the commercially available labelled nanoparticles, which under biological conditions can, in some cases, release a significant amount of labile dye, which has a completely different uptake kinetics and sub-cellular distribution compared to the nanoparticles-bound dye.[1] In these particles the dye was previously believed to be strongly entrapped into the glassy polymer network and that the diffusion time from the glass would be so long that it would not be significant over the lifetime of the particles. Although these commercial particles do not appear to leach dye in aqueous solution, once they are in contact with the hydrophobic environment of the cell a significant fraction of free dye elutes rapidly from the nanoparticles.[1], [2] This phenomenon is not unique for nanoparticles, but is also true for labelled dextrans.[3]

A similar phenomenon also occurs for nanoparticles synthesised and labelled by conventional laboratory approaches, using a labelled co-monomer and a free radical polymerisation synthesis route. We believe that the phenomenon is very widespread in nanoparticle synthesis. The example presented here uses N-isopropylacrylamide (NIPAM) nanoparticles labelled with a rhodmaine functionalised to allow its copolymerisation. Following synthesis, the nanoparticles were extensively dialysed against water to remove the surfactant molecules (which are toxic to cells) and unreacted / unbound dye or against ethanol (in which the rhodamine is well soluble) followed by dialysis in water. Polyacrylamide gel electrophoresis (PAGE) was used to monitor the elution of free dye from the nanoparticles using both types of cleaning (dialysis against water / or dialysis against ethanol then water), confirming that unreacted rhodamine continues to be released from the particles over extended time periods. Cellular uptake and sub-cellular distribution of the particles cleaned by both methods was determined using confocal microscopy and the sub-cellular localisation was determined, showing clear differences depending on the amount of free dye present as determined from the PAGE gels. Thus, PAGE analysis of the release of dye from labelled nanoparticles provides a simple and effective approach to assessing the suitability of labelled nanoparticles for cellular uptake and localisation studies, and to ensure that uptake kinetics and uptake amounts take account of the presence of free dye in many nanoparticle systems which can be mobilised and released under cell culture conditions.

## Results

**Figure 1:**
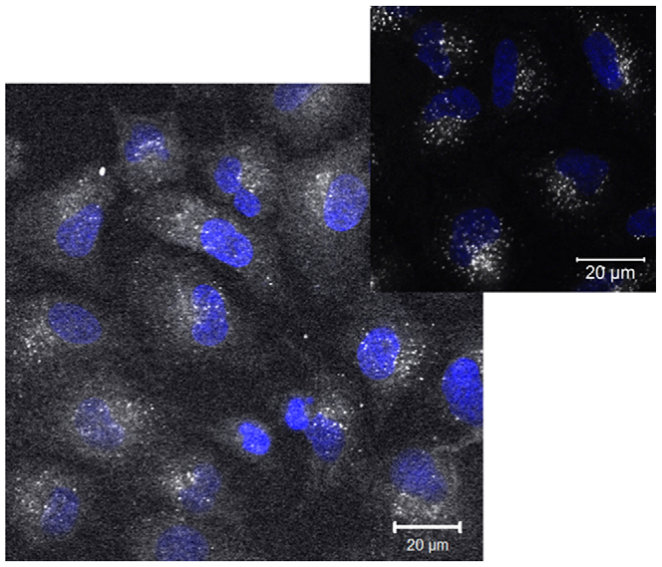
Cellular fluorescence associated with particles leaking dye versus non-leaking particles. Distribution of fluorescence in A549 cells following 24 hours of exposure to 100 µg/mL rhodamine-labelled nanoparticles cleaned by dialysis against water for 20 days. Note the distribution of the dye throughout the cytoplasm, suggesting a significant contribution from free-dye also. Inset: Distribution of fluorescence following uptake of nanoparticles cleaned by dialysis against ethanol for 7 days (60 µL sample from dialysis tube dispersed in 1.5 mL cMEM–see SI for details of the sample preparation and the determination of the particle concentration in the dialysis stock solutions)-fluorescence is localised in vesicles (likely lysosomes). Blue: DAPI stained nuclei; White: fluorescence intensity (free dye and/or nanoparticles; greyscale).

N-isopropylacrylamide (NIIPAM) nanoparticles with a fluorescent co-monomer, methacryloxyethyl thiocarbamoyl rhodamine B, were synthesised by free radical polymerisation, as described in the Experimental Details. Following synthesis, the nanoparticles were extensively dialysed against water to remove the surfactant molecules (which are toxic to cells) and unreacted / unbound dye. After 20 changes of water no further release of dye could be observed by fluorescence measurements, and the conductivity of the dialysate water was close to that of the MilliQ, indicating that the SDS was removed. Nanoparticles were characterised for size and size distribution in both water and complete Modified Eagles Medium containing 10% foetal calf serum (cMEM), and were determined to have a size of 48±20 nm at 37°C in water, as shown in Figure 1. The size of the particles increased upon dispersion in cMEM to ∼280±100 nm, likely as a result of both formation of a “corona” of proteins around the nanoparticles,[4], [5] which induces some additional interaction between the particles, and the reduction in the electrostatic repulsion resulting from the absorption of proteins and the consequent slight reduction of the zeta potential as the residual charges from the persulfate initiator are screened. However, the significant size distribution, and the tail below 100 nm suggests that at least some nanoparticles may be taken up by cells (Figure S1). The presence of rhodamine had no significant influence on the physical properties of the particles, as determined by the fact that the introduction of the rhodamine comonomer did not result in any change of the lower critical solution temperature or transition temperature of the particles (Figure S2), compared to the 33°C for pure NIPAM particles or polymers.[6], [7]

As a first step in the characterisation of the usefulness of the rhodamine-labelled nanoparticles for use in biological uptake studies, it was necessary to confirm that sufficient fluorophore was incorporated into the nanoparticles that they could be observed by confocal microscopy. Thus, the particles cleaned by dialysis against water were dispersed in cMEM and exposed to A549 cells as described in the experimental section. A549 cells were exposed to 100 µg/mL of the rhodamine labelled nanoparticles for 24 hr at 37°C under standard tissue culture conditions and the uptake was monitored using confocal microscopy. Several significant challenges in terms of labelling nanoparticles for biological use were identified by these studies. The initial observation was that the fluorescence was distributed throughout the cell, which is somewhat unexpected for nanoparticles, which are expected to be taken up by receptor mediated processes, and as such should be confined to endosomal or lysosomal type structures. A similar pattern of distribution had previously been observed by our group for commercially available fluorescent polystyrene nanoparticles, and detailed studies using confocal microscopy and flow cytometry under a range of energy-depleted conditions (at 4°C and in the presence of sodium azide) confirmed that for several of the commercial particles there was a significant proportion of free-dye that eluted from the particles under biological conditions.[1] The eluted free dye was able to diffuse throughout the whole cell, whereas the nanoparticle-bound dye was confined to lysosomes by 24 hours.[1] Thus, from the from the confocal images shown in Figure 1, it was clear that here also there were two different dye populations, the dye in the cytoplasm and dye localised in organelles. These populations were subsequently confirmed as free dye that had not been removed by the dialysis against water which diffuses across the cellular membranes and distributed throughout the cell, and dye covalently bound to the nanoparticles, which are taken into the cells by specific mechanisms such as endocytosis and therefore localise to organelles such as endosomes and lysosomes, respectively. Thus, it is clear that extensive dialysis against water was not sufficient to fully remove the unbound dye from the core of the NIPAM nanoparticles, suggesting that the fluorescent monomers are quite strongly adsorbed into the particle matrix, either via hydrophobic or electrostatic interactions or a combination of these.

Figure 1 inset shows similar nanoparticles which have been extensively dialysed against ethanol, in which the rhodamine monomer is significantly more soluble, prior to final dialysis against MilliQ water, thus the free dye can be considered to be essentially removed, for comparison. Here it is clear that the dye is confined in organelles (lysosomes), and as such that the dye is considered to be firmly bound to the nanoparticles. The processes used for monitoring and confirming the removal of free dye from the particles in the inset are described below. It is clear that the fluorescence intensity of the image in the inset is much lower than that of the main figure, captured at identical settings on the confocal microscope, showing an additional challenge in the preparation of labelled nanoparticles for cellular imaging of uptake-namely achieving sufficient fluorescence intensity whilst also ensuring that the dye is appropriately incorporated into the particles so that it does not leech out under biological conditions.

**Figure 2:**
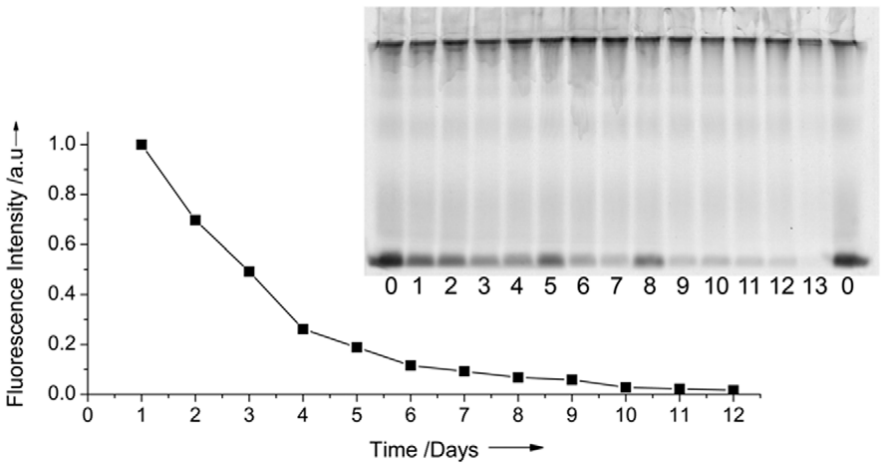
Fluorescence spectrometry analysis of the release of un-bound rhodamine dye from NIPAM nanoparticles. The curve shows the fluorescence intensity of the ethanol dialysate (samples taken every 24 hours with the ethanol changed each time). Inset: 1D-SDS PAGE of rhodamine-labelled NIPAM particles following dialysis against ethanol for increasing times. Sample 0 is the as-synthesized nanoparticles, samples 1–12 are taken sequentially every 24 hours following dialysis against a fresh bath of ethanol. Sample 13 is taken after an additional 10 days of dialysis (day 23). Note that sample 0 was loaded at the beginning and at the end of the gel to aid the eye.

Based on the data in Figure 1, it is clear that an additional cleaning process was required in order to make the rhodamine-labelled NIPAM nanoparticles suitable for use in biological uptake studies. As we were using a rhodamine monomer, we expected that the release of unbound dye would be a less significant problem than for the commercial particles, where the dye is entrapped in the particles. However, the results shown in Figure 1 suggest that not all of the rhodamine reacted and was polymerised into the particles, and that there was a significant proportion of the rhodamine simply entrapped in the polymeric network of the particles, which was insufficiently soluble in water to be removed by the dialysis process. Thus, in an effort to try to improve the removal of the free (un-reacted) rhodamine dye from the NIPAM nanoparticles, subsequent batches of rhodamine-labelled nanoparticles were dialysed extensively against ethanol, and the release of unbound dye from the nanoparticles into the dialysate was tracked using fluorescence spectroscopy, as shown in Figure 2, prior to the final dialysis against water. After about 12 days of dialysis against ethanol the release of dye from the particles was so low that it cannot be detected by fluorescence measurements.

Another approach that was used to confirm that all free dye was removed from the nanoparticles was size exclusion based on 1D polyacrylamide gel electrophoresis (1D PAGE), which was used to visualize both the nanoparticle-bound dye and any residual labile dye, and the relative proportions of each during the dialysis against ethanol. PAGE is conventionally used to separate proteins according to their molecular weight, on the grounds that smaller proteins will move faster through the gel than larger ones, in response to an electric current. The principle of the experiment shown here is that the nanoparticles are too large to enter the gel and so remain at the top of the gel, whereas the labile dye released from the particles would enter the gel, and thus be effectively separated from the nanoparticles. In order to monitor the efficacy of the dialysis against ethanol, an aliquot was removed from the nanoparticle dispersion every 24 hours, and the ethanol was replaced. After the final dialysis step, equal amounts of each sample were mixed with loading buffer, and samples were separated by PAGE as described in the experimental details. A gel image was obtained by scanning the gel with a fluorescence scanner, as shown in Figure 2 inset. The bands in the upper part of the gels represent the fluorescence from the nanoparticles which remain in the stacking gel, while the bands at the bottom of the gels are from the free rhodamine monomer, which has eluted from the nanoparticles in the lipophillic environment of the SDS/glycerol loading buffer, and is subsequently separated from the particle-bound dye on the gel due to its molecular weight. Free dye can be observed in all samples up to sample 13 (corresponding to 24 days of dialysis against ethanol), although the concentration of free dye decreases with each dialysis step.

**Figure 3:**
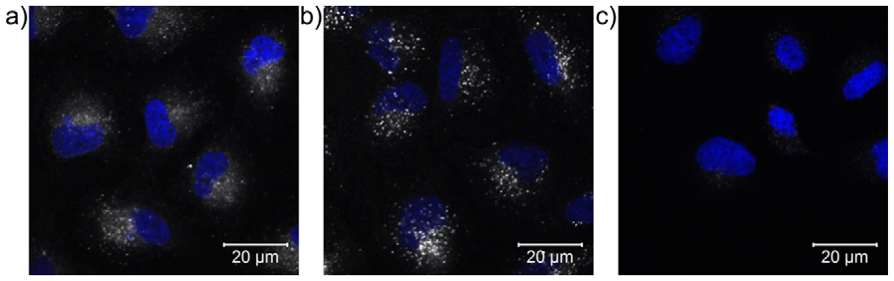
Illustration of the reduction of “free” dye following extensive dialysis of the nanoparticles against ethanol. Comparison of the distribution of fluorescence in A549 cells following 24 hours of exposure to rhodamine-labelled nanoparticles cleaned by dialysis against ethanol (60 µL sample from dialysis tube in 1.5 mL cMEM–see SI Text S1 for details) for (a) 1 day; (b) 7 days; and (c) 23 days. All images were acquired using identical settings on the confocal microscope (see SI Text S1 for details) and the imaging settings were optimised to visualise the distribution of the labelled particles dialysed against ethanol for 7 days. Images with the settings optimised for the samples cleaned for 1 and 23 days are given in Figure S4. Blue: DAPI stained nuclei; White: fluorescence intensity (free dye and/or nanoparticles; greyscale).

As well as removing the unbound dye from the nanoparticles, it is essential that the particles retain sufficient dye to enable their visualisation under confocal microscopy in order to track their interaction with cells. To this end, three nanoparticles samples taken at three different stages of cleaning (i.e. after different lengths of dialysis against ethanol, namely 0, 7 and 23 days of dialysis) and final cleaning by dialysis against Milli Q water (as ethanol is also damaging to cells) were presented to A549 cells (60 µL samples from dialysis tube in 1.5 mL cMEM) for 24 hr at 37°C under standard tissue culture conditions and their uptake and fluorescence distribution were determined using confocal microscopy, as shown in Figure 3. From these images it is clear that significant effort is needed to remove labile, physically adsorbed fluorescent labels, that have not been covalently bound into the polymeric backbone of polymeric nanoparticles in order to utilise them for biological assays, as cells themselves are extremely efficient filters for labile dyes resulting in complex uptake and distribution patterns due to the presence of nanoparticle-bound and free dye. Thus, Figure 3(a) shows the same pattern of distribution of the fluorescence as seen in Figure 1, where a significant fraction of the fluorescence is from free dye distributed throughout the cell. Figure 3(b) shows that the majority of the dye is associated with nanoparticles, which are confined in lysosomes. Figure 3(c), after several additional days of dialysis shows that the nanoparticles now contain too little fluorescence to be observed under these settings. However, it must be noted that the settings were optimized for the particles at day 7 (see below for further details) in order to make a direct comparison between the three samples. This result does show however that over-cleaning of the particles can result in the particles not being sufficiently bright to be visualized, so some compromise may be needed between removing sufficient unreacted dye so as not to compromise the uptake results, whilst allowing the particles to retain sufficient dye to be visualized by confocal microscopy. Figure S3 shows the fluorescence spectra for the three samples used in Figure 3, both as the raw data and corrected for the number of particles in the samples, as determined from Nanoparticle Tracking Analysis using NanoSight LM10 (see Supporting Information Text (SI Text S1) for full details). Figure S4 shows confocal images the same three samples where the images was acquired using settings optimised for each specific sample (see SI Text S1 for details) in order to illustrate the fact that for each particle we can image the resulting particles, irrespective of the duration of dialysis, but that in the case of too little dialysis we see fluorescence from free dye in addition to nanoparticles (Figure S3A), and in the case of the 23 days of dialysis, we have to push the laser power and the detector so much that both cellular auto-fluorescence and detector noise become a problem (Figure S3C).

## Discussion

We have shown here that 1D PAGE gels is a more reliable method than fluorescence spectroscopy to confirm the presence of, and relative amount of, free dye in nanoparticle samples and to monitor the progress of cleaning processes applied to the nanoparticles in order to render them suitable for biological assays. We suggest that this approach should be used routinely for all labeled nanoparticles, both synthesized in laboratories and commercially available, to ascertain whether particles being used in biological assays contain a fraction of labile dye. This knowledge is essential for quantification of cellular uptake of nanoparticles and their sub-cellular localization, both of which are entirely different for molecular dyes (which partition according to equilibrium principles) and nanoparticles which are actively transported by the cellular machinery. Our group has developed an approach to quantify the contribution to the overall uptake kinetics from the free and nanoparticles-bound dyes.[1]

Fluorescent nanoparticles prepared by several common synthesis routes, including copolymerization with a fluorescent monomer or entrapment of dye within a glassy polymer, contain a (significant) fraction of residual free dye that cannot be easily removed by conventional cleaning techniques such as dialysis. Consequently, the total fluorescence observed in biological uptake studies is composed of the fluorescence contribution from the free dye and that from the nanoparticle-bound dye. The proportions of each fraction, and the progress of the cleaning steps, can be easily monitored by PAGE.

This is an important message that must be disseminated to the wider nanoparticle community, since, as we have shown here, even after extensive cleaning of the nanoparticles by dialysis against a solvent in which the dye is soluble, a fraction of labile dye can remain. An additional complication is that removal of all of the labile dye can result in the particles not being sufficiently bright to be observed in cells, meaning that they are of limited use following cleaning. We are currently working on development of strategies to enhance the covalent binding of fluorescent monomers into nanoparticles during free radical polymerization.

The method presented here enables a simple characterization of the dye-integrity of nanoparticles, and can be used to determine the contribution of free dye to uptake profiles determined by confocal microscopy, flow cytometry or other fluorescence approaches. We would recommend that such a characterization be included as standard for all fluorescently-labelled nanoparticles, both commercially available samples, and those synthesized bespoke in laboratories around the world for biological applications.

## Materials and Methods

### Materials

N-isopropylacrylamide (NIPAM, Polysciences, Inc.) was purified by recrystallizing from n-hexane (Sigma-Aldrich) twice and dried *in vacuo* prior to use. The surfactant (sodium dodecyl sulfate, SDS) from BDH Chemicals LTD, the cross-linker (N,N'-methylene(bisacrylamide), BIS) from Fluka and the initiator, (ammonium persulfate, APS) from Sigma-Aldrich were all used as received. The fluorescent monomer N-[9-(2-carboxy-x-methacryloxy-ethylthiocarbamoylphenyl)-6-diethylamino-3H-xanthen-3-ylidene]-N-ethyl-ethanaminium chloride PolyFluor® 570 (methacryloxyethyl thiocarbamoyl rhodamine B) from Polysciences, Inc was used as received. Distilled water was purified with an RG MilliQ Ultra Pure Water System and filtered through a MPGL04SK2 Millipak 40, with 0.22 µm pores to remove particulate matter.

### Synthesis of rhodamine-labelled NIPAM nanoparticles

Microgel particles composed of 100% NIPAM (1.4 g) with nominally 1000 fluorescent labels per particle were synthesized by emulsion polymerization in ultra pure water (95 ml) at 70°C under the flow of inert gas (N_2_), with (N,N'-methylene(bisacrylamide), as the cross-linker (0.14 g), ammonium persulfate as the initiator (0.0475 g), sodium dodecyl sulphate as the surfactant (0.4 g) and methacryloxyethyl thiocarbamoyl rhodamine B as the fluorescent monomer (0.0088 g). The dye and the SDS were mixed in 20 ml of water and sonicated for 7 hours continuously in the ultrasonic bath (Branson 1510) at a frequency of 42 KHZ until all the dye was completely dissolved by visual inspection. The monomers plus additional 20 ml water were added to this solution obtaining a solution of 40 ml. This solution was sonicated in the ultrasonic bath continuously for 4 hours. Then the solution was divided into two solutions each one of 20 ml that were sonicated in the ultrasonic bath for 8 hours continuously until all the monomers were dissolved and after that the two solutions were combined together and the synthesis was performed by mixing all the reactants together, stirring and degassing by bubbling with N_2_ for 30 minutes while heating to 70°C. 5 ml of the degassed initiator was added to start the reaction, which was allowed to proceed for 12 hours.

It is important that the particles are dialysed extensively to remove the SDS, which is toxic to cells, and the unbound fluorescent dye in order to be able to perform quantitative cellular uptake measurements. The rhodamine-labelled nanoparticles were dialysed against water for 20 days and against ethanol (for up to 23 days) in order to remove the free dye not chemically bound and then extensively dialysed in ultra pure water as traces of ethanol in the particles would also influence the biological response observed.

### Nanoparticle characterisation

Nanoparticles were characterised for size, size distribution and their thermoresponsive characteristics in water. DLS measurements at the scattering angle θ = 173° were performed using a Malvern Zetasizer NANO ZS with a He-Ne red laser with a wavelength of 632.8 nm. Full details of the characterisation are given in the SI Text S1.

### Quantification of the release of unbound dye from the rhodamine-labelled NIPAM nanoparticles

#### Fluorescence spectroscopy quantification

Immediately following synthesis, the rhodamine-labelled NIPAM nanoparticles were transferred to cellulose membrane 8000–10000 MWCO dialysis tubing and dialysed against 1200 ml of pure ethanol to remove the unbound dye. The fluorescence intensity of the dialysate (ethanol solution) was measured every 24 hours. The ethanol dialysate was changed every 24 hours to speed up the removal of the labile dye. Fluorescence was measured on a Fluorolog-3 Spectrofluorometer HORIBA JOBIN Yvon LTD Xenon Lamp, with excitation and emission wavelengths 548 and 570, respectively, and slits set at 2.

### Polyacrylamide gel electrophoresis assessment

SDS-PAGE gels were run using a minigel apparatus (10 cm×15 cm, Biorad). Gels were prepared with a 4% stacking gel (containing 5% w/v acrylamide, 0.13% w/v N,N methylene bisacrylamide, 0.1% w/v SDS, 0.125 M Tris-HCL pH6.8, 0.1% w/v ammonium persulphate and 0.2% w/v TEMED) and 12% Acrylamide. Gels were scanned using Typhoon 9200 GE Healthcare (excitation 532 nm, emission 580 nm). The porosity of the gel ensures migration of the dye but not nanoparticles. Samples taken at each time-point of the ethanol dialysis were diluted 1∶5 in double distilled water to reduce the fluorescence intensity of the nanoparticles and were mixed with a 3X loading buffer (6% SDS, 30% glycerol) prior loading to the gel. 10 ul per sample were loaded in each well. Band intensity was detected by densitometry using Image J software.

### Uptake of rhodamine-labelled NIPAM particles by A549 cells

For confocal microscopy determination of nanoparticle uptake and distribution, 1.8×10^5^ cells were seeded onto glass coverslips inside 35 mm dishes (Greiner) and incubated for 24 h under standard tissue culture conditions (37°C, 5% CO_2_) prior to addition of particles. Nanoparticle dispersions were prepared at room temperature (samples 1, 7 and 13 from the gel shown in Figure 2, corresponding to 1, 7 and 23 days of dialysis against ethanol followed by further dialysed for 3 days against Milli-Q water) by adding 60 µL particle solutions from dialysis tube in 1.5 mL cMEM immediately prior to addition to the cells. Following particle exposure, medium was removed and all samples were washed thrice with DPBS, fixed with 4% formalin solution neutral buffered, and the nucleus stained with 4′,6-diamidino-2-phenylindole (DAPI blue), and mounted using MOWIOL before analysis.

### Confocal microscopy imaging

Images of the nanoparticles in the intracellular environment were acquired using confocal microscopy (Carl Zeiss LSM 510 UVMETA, Thornwood. NY). Samples were excited with 364 nm (blue channel, DAPI staining of the nucleus), 543 nm (red channel, nanoparticles) laser lines. The gain and offset were kept constant between samples to allow semi-quantitative comparison of the cell fluorescence intensities. Since the nanoparticles samples dialysed for different lengths of time against ethanol had different fluorescent intensities, the optimal conditions had to be established, where the particles could be detected but the background and intrinsic cellular fluorescence were minimised.

The Supporting Information Text S1 for this article contains further details on the experimental details, as well as results of the detailed size characterization of the rhodamine-labelled NIPAM nanoparticles, the fluorescence spectra of the particles after the different durations of dialysis against ethanol, the method used to calculate the particle concentrations after each of the dialysis durations, and the confocal images of the cells following uptake of the three different particles where the confocal microscope setting were optimized for each sample individually, and Figures S1, S2, S3, S4.

## Supporting Information

Figure S1 Normalized size distribution by intensity of Rhodamine B labelled NIPAM nanoparticles in water (black) and cMEM (red) T = 37°C. Inset shows the size distribution of protein clusters in cMEM at T = 37°C.
(JPG)

Figure S2 Determination of the transition temperature of the Rhodamine-labelled NIPAM nanoparticles. A. Overlay of the DLS intensity plots with increasing temperature. B. A sigmoidal fit to the hydrodynamic diameter versus temperature plot is used to indicate the transition temperature (LCST) of the rhodamine-labelled NIPAM nanoparticles (the hydrodynamic diameters were taken as the centre of the CONTIN distributions shown in Figure S2A).
(JPG)

Figure S3 Emission spectra of sample 1 (1 day of dialysis), sample 7 (7 days of dialysis) and sample 13 (23 days of dialysis) determined using the Fluorolog spectrophotometer. Left: raw data; Right: emission spectra corrected for the particle concentration in each sample, as determined by NanoSight LM10 Particle Tracking Analysis.
(JPG)

Figure S4 Comparison of the distribution of fluorescence in A549 cells following 24 hours of exposure to rhodamine-labelled nanoparticles cleaned by dialysis against ethanol for (A) 1 day; (B) 7 days; and (C) 23 days. Samples were prepared by taking 60 µL sample from dialysis tube and adding it to 1.5mL cMEM, and particle concentrations were determined subsequently. Each of the images was acquired using settings optimised for that specific sample (see above for details) in order to illustrate the fact that for each particle we can image the resulting particles, irrespective of the duration of dialysis, but that in the case of too little dialysis we see fluorescence from free dye in addition to nanoparticles (A), and in the case of the 23 days of dialysis, we have to push the laser power and the detector so much that both cellular auto-fluorescence and detector noise become a problem (C).
(JPG)

Text S1 (DOC)
