# Supplementary figures and images for: Elution of Labile Fluorescent Dye from Nanoparticles during Biological Use
Source: PLoS One. 2011 Oct 6;6(10):e25556. doi: 10.1371/journal.pone.0025556 (PMC3188558; doi:10.1371/journal.pone.0025556)

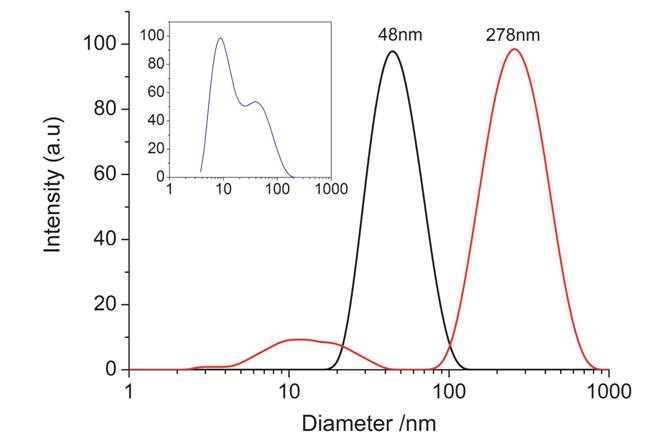

Supplement: Figure S1 — Normalized size distribution by intensity of Rhodamine B labelled NIPAM nanoparticles in water (black) and cMEM (red) T = 37°C. Inset shows the size distribution of protein clusters in cMEM at T = 37°C. (JPG) [file pone.0025556.s001.jpg]

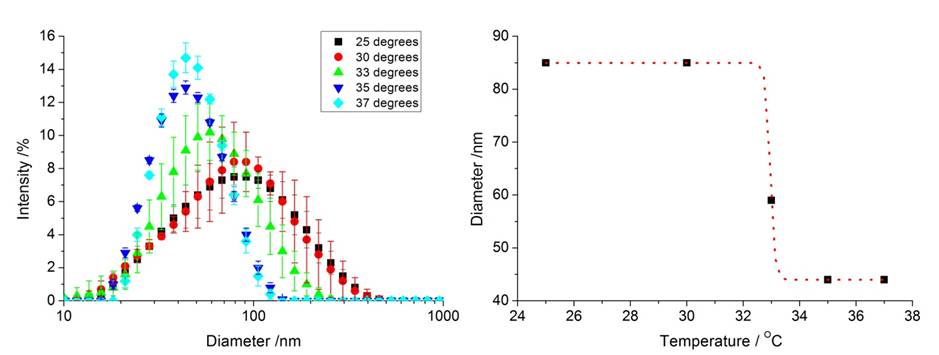

Supplement: Figure S2 — Determination of the transition temperature of the Rhodamine-labelled NIPAM nanoparticles. A. Overlay of the DLS intensity plots with increasing temperature. B. A sigmoidal fit to the hydrodynamic diameter versus temperature plot is used to indicate the transition temperature (LCST) of the rhodamine-labelled NIPAM nanoparticles (the hydrodynamic diameters were taken as the centre of the CONTIN distributions shown in Figure S2A). (JPG) [file pone.0025556.s002.jpg]

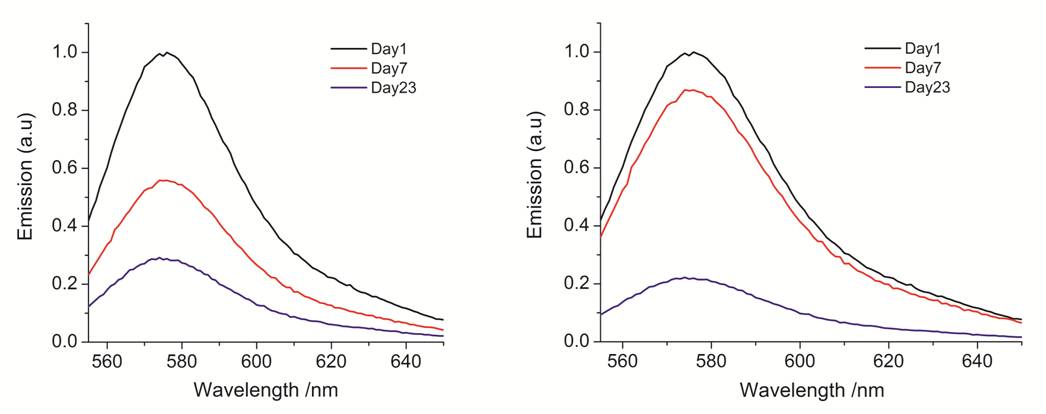

Supplement: Figure S3 — Emission spectra of sample 1 (1 day of dialysis), sample 7 (7 days of dialysis) and sample 13 (23 days of dialysis) determined using the Fluorolog spectrophotometer. Left: raw data; Right: emission spectra corrected for the particle concentration in each sample, as determined by NanoSight LM10 Particle Tracking Analysis. (JPG) [file pone.0025556.s003.jpg]

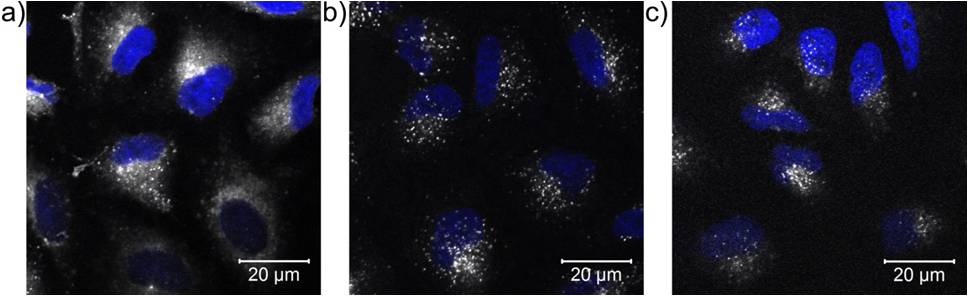

Supplement: Figure S4 — Comparison of the distribution of fluorescence in A549 cells following 24 hours of exposure to rhodamine-labelled nanoparticles cleaned by dialysis against ethanol for (A) 1 day; (B) 7 days; and (C) 23 days. Samples were prepared by taking 60 µL sample from dialysis tube and adding it to 1.5mL cMEM, and particle concentrations were determined subsequently. Each of the images was acquired using settings optimised for that specific sample (see above for details) in order to illustrate the fact that for each particle we can image the resulting particles, irrespective of the duration of dialysis, but that in the case of too little dialysis we see fluorescence from free dye in addition to nanoparticles (A), and in the case of the 23 days of dialysis, we have to push the laser power and the detector so much that both cellular auto-fluorescence and detector noise become a problem (C). (JPG) [file pone.0025556.s004.jpg]
