# Supplementary material for: Elution of Labile Fluorescent Dye from Nanoparticles during Biological Use
Source: PLoS One. 2011 Oct 6;6(10):e25556. doi: 10.1371/journal.pone.0025556 (PMC3188558; doi:10.1371/journal.pone.0025556)
Supplement: Text S1 — (DOC) [file pone.0025556.s005.doc]

*Supplementary Text*

Elution of labile fluorescent dye from nanoparticles during biological use

Tiziana Tenuta et al.

**Materials and Methods – additional information**

*Preparation of Complete MEM*

MEM was purchased from GIBCO (31045) composed of EARLE’S and L^-^ Glutamine; 1% of non essential Amino Acid (purchased from Thermo Scientific) plus 1% of PENSTRA (penicillin-streptomycin, purchased from GIBCO 15070) and 10% of Foetal Bovine Serum (purchased from GIBCO 10270-106) were added obtaining the final solution.

*Sample preparation in water (Size characterisation measurements)*

A sample was prepared by dissolving 50μl of extra cleaned NIPAM fluorescent nanoparticles, taken directly from the dialysis tubing following 23 days of dialysis in ethanol followed by 25 days of dialysis against Milli-Q water, in 5ml of Milli-Q water. This sample was sonicated in an ultrasonic bath (Branson 1510) at a frequency of 42 KHZ for 15 minutes then left at 4 °C for 12 hours. Before measurement the sample was left at room temperature for 1 hour, degassed and filtered with 0.2 μm cellulose acetate filters suitable for aqueous solutions in order to remove dust or any impurities contained in the Milli-Q water and then left for 3 hours at the measurement temperature in order to give the system the necessary time to stabilize.

*Sample preparation in Complete MEM (size characterisation measurements)*

A sample was prepared dissolving 50μl of extra cleaned NIPAM fluorescent nanoparticles, taken directly from the dialysis tubing following 23 days of dialysis in ethanol followed by 25 days of dialysis against Milli-Q water, in 5ml of Complete MEM. This sample was shaken for few minutes using a vortex then incubated at 4°C for 1 hour. Immediately after incubation the sample was degassed and left for 2 hours at the measurement temperature (T = 37°C) for further stabilization, prior to measurement.

*Characterization of the rhodamine-labelled NIPAM nanoparticles by Dynamic Light Scattering*

*Temperature Dependence of Size & Size Distribution in Water:*

DLS measurements at the scattering angle θ = 173° were performed using a Malvern Zetasizer NANO ZS with a He-Ne red laser with a wavelength of 632.8 nm. The maximum excitation (548nm) and emission (570nm) of the red fluorescent monomer did not interfere with the DLS measurements. A sample in water was prepared as above. Each DLS measurement was an average of eleven repetitions of one minute each and repeated six times maintaining the temperature at T = 37°C.

Data analysis was performed according to standard procedures and interpreted through a cumulant expansion of the field autocorrelation function to the second order. Moreover, in order to obtain a distribution *w(Γ)* of decay rates (converted to *w(R_H_)*), a constrained regularization method, CONTIN, was used to invert the experimental data. The normalized size distribution by intensity of Rhodamine B labelled NIPAM nanoparticles in water and cMEM at 37°C is shown in Figure S1.

The transition temperature of the rhodamine-labelled NIPAM nanoparticles was also determined by Dynamic Light Scattering. A sample in water was prepared as above and DLS measurements were performed at different temperatures. The temperature was increased by few degrees starting from T = 25 °C until T = 37 °C, allowing the sample to stabilize at each temperature for about 30 minutes. The hydrodynamic diameter at each temperature (reported in Figure S2B) were taken as the centre of the CONTIN size distribution. Each measurement was an average of 11 repetitions and at least four runs (Figure S2A). The standard deviation is shown as error bars in Figure S2A.

*Optimisation of the setting for confocal microscopy imaging*

Images of the nanoparticles in the intracellular environment were acquired using confocal microscopy (Carl Zeiss LSM 510 UVMETA, Thornwood. NY). Samples were excited with 364 nm (blue channel, DAPI staining of the nucleus), 543 nm (red channel, nanoparticles) laser lines. The gain and offset were kept constant between samples to allow semi-quantitative comparison of the cell fluorescence intensities. Since the nanoparticles samples dialysed for different lengths of time against ethanol had different fluorescent intensities, the optimal conditions had to be established, where the particles could be detected but the background and intrinsic cellular fluorescence were minimised. The optimised confocal settings used for the three samples were as follows:

- Sample 1: Red channel: gain 668 / offset 0.012; Blue channel: gain 864 / offset 0.005.
- Sample 8: Red channel: gain 688 / offset 0.022; Blue channel: gain 954 / offset 0.
- Sample 14: Red channel: gain 898 / offset 0.027; Blue channel: gain 954 / offset 0.

*Quantification of the concentration of NIPAM nanoparticles*

As the samples used for the confoal microscopy imaging (Figure 3 and Figure S4) and for the comparative fluorescence intensity study (Figure S3) were taken as aliquots from the dialysis tubing, their exact particle concentration was unknown. In order to obtain an approximation of the actual concentrations and to ensure that the fluorescence intensities are comparable in terms of particle numbers, the particle concentration was determined using NanoSight LM10 Nanoparticle Tracking Analysis, according to the method described in the NanoSight Technical Note on Concentration Measurements.[1]

For one of the three time-points (sample 13 corresponding to day 23 of dialysis against ethanol) three different time-lapse videos were made, and for each video three different measurements were made, and the results were averaged. For the other two samples (day 1, day 7) one video was made with 3 different measurements, and the results were averaged.

**Supplementary Results and Discussion**

*Nanoparticle characterisation*

The size was determined in water and in Complete MEM at T = 37 °C and the results are contained in Figure S1. These measurements showed a good size stability and good sample monodispersity, as shown in Figure S1, with a significant increase in the size in cMEM compared to water. There is a clear increase in the size of the particles in cMEM compared to the size in water, which is indicative of some interaction between the particles and formation of clusters of approximately 3 particles and the formation of a protein corona around the particles.

The overlay of the size distributions in Figure S2B shows the gradual transition from expanded (polymer-water contacts are favourable) to collapsed (polymer-polymer interactions are favourable) particles. A sigmoidal fit to the hydrodynamic diameter versus temperature plot (Figure S2B) gives the value of 33 °C as the inflection point, showing that the thermo-responsive behaviour and the transition temperature of the fluorescent labelled NIPAM nanoparticles is not affected by the presence of the fluorescent co-monomer, and is in good agreement with the literature values of ~34 °C.[1] Thus, the presence of the Rhodamine B does not interfere with the Lower Critical Solution Temperature (LCST). The size distribution becomes narrower with increasing solution temperature (Figure S2A), showing a good sample monodispersity that makes these nanoparticles useful for nano-biological studies in human cells.

*Fluorescence characterisation of the rhodamine-B labelled NIPAM nanoparticles*

Figure S3 shows the fluorescence values of sample 1 (taken after 24 hours of dialysis in ethanol), sample 7 (taken after 7 days of dialysis in ethanol), and sample 13 (taken after 23 days of dialysis in ethanol). The fluorescence of the nanoparticles decreases over time, as more of the unbound dye is washed out of the particles. Sample 13 is the least fluorescent sample and the cleanest, in agreement with the SDS PAGE gel shown in Figure 2 in the manuscript.

*Determination of nanoparticle number in the samples presented to cells*

The approximate numbers of particles per volume in the three samples following dialysis against ethanol were determined to be:

- day 1 (sample 1): 0.7 x 10^8^ particles /ml
- day 7 (sample 7): 0.45 x 10^8^ particles /ml
- day 23 (sample 13): 0.92 x 10^8^ particles /ml

Thus, it is important to note that the concentration of particles in the day 7 sample, for which the confocal microscopy settings were optimised for the images shown in Figure 3 in the manuscript, is significantly lower than those of the other two samples. This will also be noted in the discussion later regarding the confocal images.

*Uptake of rhodamine-labelled nanoparticles by A549 cells*

The data shown in Figure 3 in the manuscript are the uptake based on optimisation of the fluorescence intensity of the particles dialysed for 7 days (which as mentioned above has the lowest particle concentration, with 0.45 x 10^8^ particles /ml). It is clear that the fluorescence from the 7 day dialysis sample is localised in vesicle-like structures, likely lysosomes. At these settings, very little fluorescence can be observed from the particles dialysed for 23 days, despite the higher number of particles. This illustrates a key challenge for particle synthesis – over cleaning the particles can result in the fluorescence being too low to be observed under confocal microscopy, and can mean that the instrument settings have to be amplified such that interference from cellular auto-fluorescence become an issue (Figure S4C). In order to visualise the fluorescence of the particles in this case, the detector gain would have to be increased which results in the generation of too much undesired background, as shown in Figure S4C. On the other hand, the particles dialysed for 1 day show significant fluorescence distributed through the cell indicating that there is also some leakage of labile dye from the nanoparticles which ends up in the cytoplasm due to diffusion and equilibrium distribution (Figure S4A).

*References*

[1] NanoSight: <http://www.nanosight.com/files/uploaded/M500B_NanoSight Technical Note _>ConcentratioMeasurements.pdf

[2] Hsiue GH, Hsu SH, Yang CC, Lee SH, Yang IK. (2002) Preparation of controlled release ophthalmic drops, for glaucoma therapy using thermosensitive poly-N-isopropylacrylamide. Biomaterials, 23: 457-462.
